# Causal relationship between basal metabolic rate and kidney stone disease: from discovery in US NHANES to evidence in UK Biobank cohorts

**DOI:** 10.1097/JS9.0000000000002658

**Published:** 2025-06-06

**Authors:** Yushu Chen, Jiahao Zhang, Zhibiao Li, Yihua Zhan, Zhicheng Tang, Juan Wang, Zhaohui He, Fucai Tang

**Affiliations:** a Department of Urology, The Eighth Affiliated Hospital of Sun Yat-sen University, Shenzhen, Guangdong, China; b Department of Urology, Sun Yat-sen Memorial Hospital of Sun Yat-sen University, Guangzhou, Guangdong, China

**Keywords:** basal metabolic rate, cross-sectional study, kidney stone disease, prospective cohort study, UK Biobank, US NHANES

## Abstract

**Background:**

Our previous genome-wide association study (GWAS) suggested a potential negative causal relationship between basal metabolic rate (BMR) and kidney stone disease (KSD) from a genetic standpoint. This study aimed to further investigate their association from a clinical etiological perspective across diverse global populations and to elucidate their dose-response relationship.

**Methods:**

A total of 21 140 adults from the US NHANES (2007–2020) and 289 007 participants without a prior history of KSD from the UK Biobank (2006–2024) were analyzed using cross-sectional and prospective cohort study designs, respectively. Data were collected through questionnaires and physical examinations. Multivariable logistic regression models and restricted cubic spline (RCS) analyses were employed to assess the relationship between BMR and the risk of KSD, adjusting for potential confounders. Subgroup and sensitivity analyses were conducted to explore gender- and age-related differences and evaluate the robustness of the results.

**Results:**

In the NHANES cohort, the fully adjusted odds ratio (OR) for the highest BMR quartile (Q4) compared to the lowest quartile (Q1) was 0.49 (95% CI: 0.35, 0.70; *P* for trend < 0.001). During the follow-up period in the UK Biobank cohort, 3620 participants ultimately developed KSD, and the relevant analysis further confirmed this negative causal association, with an OR of 0.72 (95% CI: 0.59, 0.89; *P* for trend = 0.003). In both cohorts, higher BMR was associated with a decreased risk of KSD, with consistent trends observed across sex and age subgroups. Sensitivity analyses validated the robustness of these findings.

**Conclusions:**

In conclusion, a higher BMR appears to be a protective factor against KSD, with a negative causal association identified. Lifestyle interventions aimed at increasing BMR may help prevent the development of kidney stones.

## Introduction

Basal metabolic rate (BMR), the minimum energy expenditure required to sustain essential life functions at rest, accounts for approximately 60–70% of daily energy consumption, underscoring its pivotal role in overall energy metabolism^[1]^. Factors influencing BMR include age, sex, body composition, and muscle mass^[2,3,4]^. A gradual decline in BMR with age is primarily attributed to the reduction in muscle mass^[3]^. Recent studies have highlighted the relevance of BMR in various health metrics, such as blood pressure^[5]^, triglycerides^[6]^, blood glucose^[7]^, and waist circumference^[8]^. Furthermore, a lower BMR has been identified as a predictor of obesity and related metabolic complications^[9]^, emphasizing the importance of strategies to increase the level of BMR^[10]^.

**HIGHLIGHTS**

- This study investigates the causal relationship between basal metabolic rate (BMR) and kidney stone disease (KSD), using data from both the retrospective US NHANES cohort and the prospective UK Biobank cohort.
- The US NHANES cohort (n = 21 140) initially identified an inverse association between BMR and KSD risk, suggesting BMR to be a potential protective factor.
- Prospective validation in the UK Biobank cohort (n = 289 007) confirmed the causal nature of this relationship, demonstrating consistent results after excluding individuals with baseline KSD.
- This study provides novel insights into the role of metabolic activity in KSD prevention and suggests that interventions targeting BMR could help reduce the risk of kidney stone formation in high-risk populations.

Kidney stone disease (KSD), also known as nephrolithiasis, is among the most common urological disorders, with incidence rates reaching up to 14.8% and rising, particularly in developed countries^[11,12,13]^. The risk factors for KSD can be influenced by certain metabolic-related dietary components, such as sugar, domestic water, meat, and salt intake^[14,15,16]^. Due to its multifactorial etiology involving dietary, lifestyle, metabolic, and genetic factors, the recurrence rate within 5 years of an initial stone episode can be as high as 50%^[17,18,19]^. Despite advancements in medical and surgical treatments for nephrolithiasis over recent decades^[17]^, its high incidence and recurrence rates contribute to substantial morbidity and health care costs. These include severe pain, urinary tract obstruction, and an elevated risk of chronic kidney disease (CKD)^[11,12]^. Therefore, understanding the underlying etiology of KSD is essential for developing effective prevention strategies and improving patient outcomes.

Although substantial literature exists on the risk factors for KSD, such as diet, fluid intake, genetic predisposition, hypertension, and metabolic disturbances, including obesity, insulin resistance, and dyslipidemia^[11,12,18-20]^, the link between KSD and BMR, which has received limited attention, represents a relatively novel area of research. Previously, only one retrospective study suggested that BMR might serve as a risk marker for predicting stone recurrence in obese patients with KSD^[21]^. In contrast, our prior genetic evidence from a genome-wide association study (GWAS) preliminarily indicated a negative causal correlation between BMR and the risk of KSD from a genetic perspective^[22]^.

Given these conflicting findings, large-scale, population-based studies are needed to further investigate this potential link and elucidate the underlying mechanisms. Therefore, we aim to utilize two large independent cohorts – the US National Health and Nutrition Examination Survey (US NHANES) and the UK Biobank – to evaluate the complex relationship between BMR and incident KSD. These databases offer a unique opportunity to explore this relationship in large, diverse populations with detailed health, dietary, and lifestyle information^[23]^. By leveraging these extensive datasets, we seek to determine whether higher BMR is protective against kidney stone formation and provide additional insights into the potential mechanisms involved. This research will enhance the existing body of knowledge by employing a comprehensive approach and may have significant implications for the prevention and management of nephrolithiasis.

## Methods

### Study design and population

The current study employed a representative cross-sectional survey (US NHANES) and a prospective cohort study (UK Biobank) to explore the relationship between BMR and the risk of KSD. The inclusion of two large-scale datasets facilitated a comprehensive analysis across diverse populations, enhancing the robustness and generalizability of the findings. This study was conducted in accordance with the principles of the Declaration of Helsinki and was registered in the Research Registry (https://www.researchregistry.com/) under the unique identifying number: researchregistry11226. Moreover, it has been reported in line with the latest version (2025) of the Strengthening the Reporting of Observational Studies in Epidemiology (STROCSS) guidelines^[24]^.

US NHANES: The US NHANES is a complex, multistage probability sampling survey conducted by the National Center for Health Statistics (NCHS) to assess the health and nutritional status of adults and children in the United States^[25]^. Data from 2007 to 2020 were utilized, focusing on adults aged 20 years and older. A total of 66 148 subjects were initially identified. After excluding participants with missing data on self-reported kidney stone history, BMR-related data, and other key covariates, 21 140 individuals were included in the analysis (Supplemental Digital Content, Figure 1, available at: http://links.lww.com/JS9/E283). The NHANES datasets are publicly available at https://wwwn.cdc.gov/nchs/nhanes/default.aspx and were approved by the NCHS Research Ethics Review Board. All participants provided informed consent before enrollment (https://www.cdc.gov/nchs/nhanes/about/erb.html?CDC_AAref_Val=https://www.cdc.gov/nchs/nhanes/irba98.htm). UK Biobank: The UK Biobank is a large, population-based prospective cohort that enrolled more than 500 000 participants aged 37–73 years from England, Scotland, and Wales during the recruitment period from 2006 to 2010^[26,27]^. To ensure comparability with the NHANES dataset, we excluded participants with incomplete data on kidney stone diagnosis, BMR measurements, and relevant covariates. Furthermore, those with a history of KSD at baseline were excluded to establish causal temporality, resulting in a final analytic cohort of 289 007 individuals (Supplemental Digital Content, Figure 1, available at: http://links.lww.com/JS9/E283). The UK Biobank datasets are available at https://www.ukbiobank.ac.uk, subject to registration and application process (Application number 143798). It received full ethical approval from the North West Multi-Center Research Ethics Committee, the National Information Governance Board for Health and Social Care in England and Wales, and the Community Health Index Advisory Group in Scotland (REC reference: 21/NW/0157; IRAS project ID: 299116). All participants provided informed consent before enrollment (http://www.ukbiobank.ac.uk/ethics/).

### Exposure and outcome assessment

Exposure variable: BMR was the primary exposure variable.

In the US NHANES, BMR was estimated using the Mifflin-St Jeor equation, which calculates BMR based on age, sex, weight, and height^[28,29]^. The formula is as follows: For men: BMR (kcal/d) = 9.99 × weight (kg) + 6.25 × height (cm) − 4.92 × age (years) + 5.

For women: BMR (kcal/d) = 9.99 × weight (kg) + 6.25 × height (cm) − 4.92 × age (years) − 161

In the UK Biobank, BMR was measured using an impedance device (Tanita BC418MA body composition analyzer), following a standardized body composition measurement protocol (Resource 1421) at an assessment center of the UK Biobank (a temperature- and humidity-controlled laboratory). This procedure ensured accurate and consistent measurements across all participants.

Outcome variable: The presence of KSD served as the main outcome. In the US NHANES, participants who answered “yes” to the question, “Have you ever had kidney stones?” were classified as having a history of KSD. In the UK Biobank, KSD diagnoses were identified from hospitalization records using the International Classification of Diseases, Ninth and Tenth Revisions (ICD-9 and ICD-10), as well as the Office of Population Censuses and Surveys Classification of Interventions and Procedures Versions 3 and 4 (OPCS-3 and OPCS-4)^[14]^ (Supplemental Digital Content, Table 1, available at: http://links.lww.com/JS9/E284). Additionally, self-reported surgical histories and death records from the calculus of the kidney and ureter were also included for KSD diagnosis. The study period spanned from the baseline date to the end of the follow-up. The follow-up endpoint was defined as the time of KSD diagnosis, participant death, or the deadline of this study (27 April 2024).

### Covariates

A range of covariates was selected based on previous research as potentially related to both BMR and KSD. These covariates were included to ensure comprehensive adjustment for potential confounders and to minimize bias in the analysis. Due to differences in data collection methods between the two cohorts, efforts were made to harmonize the definitions and classifications of covariates, ensuring consistency and comparability. Where necessary, recognized methodologies or standard definitions from each database were applied. The specific covariates and their classifications are as follows:

Demographic variables^[3,30]^: Age (≤50 and >50 years), sex (male and female), race (white and non-white), education level (≤high school and >high school), and income level (low, medium, and high).

To be specific, age was treated as a continuous variable in both datasets and categorized as age ≤50 years and age >50 years. Sex was categorized as male and female in both NHANES and UK Biobank. For race, participants were classified as White and Non-White in NHANES (Hispanic, Non-Hispanic White, Non-Hispanic Black, and Other) and UK Biobank (Asian, White, Black, Mixed, and Other). In NHANES, income level was based on the Family Poverty to Income Ratio (FPIR), with participants categorized as low (FPIR≤1), medium (1 < FPIR<4), and high (FPIR≥4). In the UK Biobank, income information was collected via a touchscreen question, “What is your average pre-tax total household income?” Participants were divided into three categories: low income (<£18 000), medium income (£18 000–£30 999 and £31 000–£51 999), and high income (≥£52 000 and >£100 000).

Lifestyle variables^[6,31]^: Smoking status (never, previous, and current), alcohol consumption (more and less), physical activity (active and inactive), and sleep duration.

Specifically, smoking status was assessed through the questions, “Have you ever smoked at least 100 cigarettes?” and “Do you currently smoke?” in NHANES. Participants were classified as never smokers, former smokers, and current smokers. Alcohol consumption was assessed with the question, “How often do you drink alcohol in the past 12 months?” Participants who drank once or more per month were categorized as regular drinkers, and those who drank less than once per month or never drank were categorized as non-regular drinkers. Similarly, the UK Biobank used structured questionnaires to categorize smoking status and alcohol consumption based on past and current frequency. In NHANES, physical activity was assessed using the Global Physical Activity Questionnaire (GPAQ), with participants categorized as active and inactive based on their self-reported levels of vigorous or moderate physical activities (e.g., running, basketball, brisk walking, or bicycling). In the UK Biobank, physical activity was assessed according to the 2017 UK Physical Activity Guidelines, with participants classified as active if they met the criteria of at least 150 minutes of moderate-intensity or 75 minutes of vigorous-intensity activity per week. Those who did not meet these criteria were categorized as inactive. In both NHANES and the UK Biobank, sleep duration was recorded in hours and treated as a continuous variable.

Health conditions^[5,6,11,32]^: Hypertension, diabetes, hyperlipidemia, body mass index (BMI), and waist circumference.

In the US NHANES cohort, hypertension was defined as either a self-reported diagnosis, the use of antihypertensive medication, or measured elevated blood pressure (systolic ≥140 mmHg or diastolic ≥90 mmHg) levels. Diabetes was defined by a self-reported diagnosis, the use of antidiabetic medications, or elevated fasting glucose (≥126 mg/dL) or glycohemoglobin (≥6.5%) levels. Hyperlipidemia was defined by a self-reported diagnosis, the use of lipid-lowering medications, or abnormal cholesterol (≥240 mg/dL), triglyceride (≥200 mg/dL), high-density lipoprotein (male <40 mg/dL, female <50 mg/dL), or low-density lipoprotein (≥160 mg/dL) levels. In the UK Biobank cohort, the histories of hypertension, diabetes, and hyperlipidemia were identified using the ICD-9 and ICD-10 codes. BMI was computed using the formula weight (kg)/(height (m)^2^) and classified into three categories: <25 kg/m^2^, 25–29.9 kg/m^2^, and ≥30 kg/m^2^. Waist circumference was measured in centimeters (cm) and treated as a continuous variable.

### Statistical analysis

Descriptive Analysis: Descriptive statistics were used to summarize the baseline characteristics of participants in both cohorts. Categorical variables were presented as counts and weighted percentages (for the US NHANES) or as percentages (for the UK Biobank), while continuous variables were expressed as means with standard deviations. Comparisons between groups, categorized by KSD status and BMR quartiles, were performed using chi-square tests for categorical variables and analysis of variance (ANOVA) for continuous variables.

Multivariate analysis: Multivariate logistic regression models were employed to analyze the relationship between BMR and the risk of KSD, adjusting for all covariates mentioned above in a stepwise manner. BMR quartiles (Q1–Q4) were used to categorize participants, with Q1 serving as the reference group. RCS regression models were used to investigate the dose-response relationship between BMR and KSD risk, with knots set at the 25th, 50th, and 75th percentiles of BMR. A *P* value for non-linearity was calculated to assess the nature of the dose-response relationship.

Subgroup analysis: Subgroup analyses were conducted to determine whether the relationship between BMR and KSD risk differed across population subgroups. The analyses were stratified by sex (male and female) and age (≤50 and >50 years). These subgroup analyses helped assess potential effect modifications by these variables. In particular, a BMI subgroup interaction analysis was conducted to evaluate the association between BMR and KSD risk across BMI categories. The cohorts were divided into three BMI subgroups: <25, 25–29.9, and ≥30. We then assessed the interaction between BMI and BMR within these subgroups using multiplication interaction based on the *F*-test.

Sensitivity analysis: Several sensitivity analyses were performed to ensure the robustness of the results. First, participants with specific health conditions, such as renal failure, hypercalcemia, increased levels of vitamin D, hyperuricemia, thyroid disease, or cancer, were excluded separately to minimize their influence on kidney stone formation or BMR levels^[33,34,35]^. Second, individuals diagnosed with KSD within 3 years of follow-up were excluded to reduce the possibility of reverse causation; however, this analysis could not be performed for NHANES due to the absence of follow-up data. Third, the analysis in NHANES was restricted to individuals aged 37–73 years to align with the age distribution of the UK Biobank. This sensitivity analysis was only performed in NHANES to address concerns about age-related variations in BMR and KSD risk. Finally, the Harris–Benedict formula^[36,37]^ was used to recalculate BMR in both datasets, and extreme anthropometric values were excluded to ensure comparability across studies.

Statistical Software: All statistical analyses were performed using R software (version 4.2.1), with a significance threshold of *P* < 0.05. Survey weights were applied to NHANES data to account for the complex sampling design, ensuring population-level inferences and representative estimates of the US population^[25]^. The sample size of this study was sufficient to achieve a statistical power of 80% in the primary analysis.

## Result

### Baseline characteristics of study population

Baseline characteristics of the study population in both US NHANES and UK Biobank cohorts were categorized by KSD status (Table 1) and BMR quartiles (Supplemental Digital Content, Table 2, available at: http://links.lww.com/JS9/E285). Among the 21 140 participants in US NHANES (mean age: 46.14 years; 51.01% male), 1923 had kidney stone disease. In the UK Biobank, 3620 participants were diagnosed with KSD out of 289 007 individuals (mean age: 55.17 years; 48.43% male). Across both cohorts, adults with KSD were more likely to be older and male. They also exhibited significantly higher obesity-related parameters (BMI and waist circumference), unhealthy lifestyle factors (lower physical activity levels and higher smoking and alcohol consumption), and a greater prevalence of metabolic syndrome-related diseases (hypertension, diabetes, and hyperlipidemia).

**Table 1:** Baseline characteristics of study participants from the US NHANES and UK Biobank according to KSD status

Characteristics	US NHANES				UK Biobank			
	Total population, n = 21 140	No KSD, n = 19 217	With KSD, n = 1923	*P* value	Total population, n = 289 007	No KSD, n = 285 387	With KSD, n = 3620	*P* value
Age (years), mean (SD)	46.14 (16.44)	45.56 (16.44)	51.63 (15.43)	<0.001	55.17 (8.06)	55.16 (8.06)	55.96 (8.07)	<0.001
Sex				0.010				<0.001
Female	9992 (48.94)	9176 (49.44)	816 (44.18)		149 049 (51.57)	147 899 (51.82)	1150 (31.77)	
Male	11 148 (51.06)	10 041 (50.56)	1107 (55.82)		139 958 (48.43)	137 488 (48.18)	2470 (68.23)	
Race				<0.001				0.376
Non-White	11 775 (30.02)	10 934 (30.85)	841 (22.09)		13 363 (4.62)	13 184 (4.62)	179 (4.94)	
White	9365 (69.98)	8283 (69.15)	1082 (77.91)		275 644 (95.38)	272 203 (95.38)	3441 (95.06)	
Education level				0.777				<0.001
≤High School	8629 (33.86)	7861 (33.90)	768 (33.50)		147 911 (51.18)	145 932 (51.13)	1979 (54.67)	
>High School	12 511 (66.14)	11 356 (66.10)	1155 (66.50)		141 096 (48.82)	139 455 (48.87)	1641 (45.33)	
Income level				0.169				0.001
Low	4036 (12.57)	3699 (12.73)	337 (11.13)		45 243 (15.65)	44 597 (15.63)	646 (17.85)	
Medium	10 876 (46.11)	9848 (45.89)	1028 (48.27)		152 841 (52.88)	150 949 (52.89)	1892 (52.27)	
High	6228 (41.31)	5670 (41.39)	558 (40.60)		90 923 (31.46)	89 841 (31.48)	1082 (29.89)	
BMI (kg/m^2^)				<0.001				<0.001
<25	6102 (29.78)	5724 (30.77)	378 (20.42)		102373 (35.42)	101 512 (35.57)	861 (23.78)	
25-29.9	6955 (33.02)	6316 (33.19)	639 (31.44)		123 421 (42.71)	121 837 (42.69)	1584 (43.76)	
≥30	8083 (37.20)	7177 (36.05)	906 (48.13)		63 213 (21.87)	62 038 (21.74)	1175 (32.46)	
WC (cm), mean (SD)	99.30 (16.52)	98.73 (16.39)	104.74 (16.71)	<0.001	89.66 (13.24)	89.58 (13.21)	95.75 (13.49)	<0.001
Sleep duration (hours), mean (SD)	7.20 (1.39)	7.21 (1.38)	7.14 (1.44)	0.109	7.16 (1.02)	7.16 (1.02)	7.11 (1.15)	0.003
Physical activity				<0.001				0.005
Inactive	101 02 (41.78)	9058 (41.12)	1044 (48.10)		133 254 (46.11)	131 500 (46.08)	1754 (48.45)	
Active	11 038 (58.22)	101 590 (58.88)	879 (51.90)		155 753 (53.89)	153 887 (53.92)	1866 (51.55)	
Smoke status				<0.001				<0.001
Never	10 960 (53.04)	10 061 (53.53)	899 (48.45)		163 230 (56.48)	161 353 (56.54)	1877 (51.85)	
Previous	5334 (26.23)	4725 (25.67)	609 (31.55)		98 440 (34.06)	97 174 (34.05)	1266 (34.97)	
Current	4846 (20.73)	4431 (20.80)	415 (20.00)		27 337 (9.46)	26 860 (9.41)	477 (13.18)	
Drink consumption				<0.001				<0.001
Less	7132 (29.61)	6351 (28.89)	781 (36.38)		44 705 (15.47)	44 015 (15.42)	690 (19.06)	
More	14 008 (70.39)	12 866 (71.11)	1142 (63.62)		244 302 (84.53)	241 372 (84.58)	2930 (80.94)	
Hypertension				<0.001				<0.001
No	12 681 (64.28)	11 783 (65.87)	898 (49.26)		271 986 (94.11)	268 729 (94.16)	3257 (89.97)	
Yes	8459 (35.72)	7434 (34.13)	1025 (50.74)		17 021 (5.89)	16 658 (5.84)	363 (10.03)	
Diabetes				<0.001				<0.001
No	17 887 (88.31)	16 458 (89.34)	1429 (78.51)		277 173 (95.9)	273 872 (95.97)	3301 (91.19)	
Yes	3253 (11.69)	2759 (10.66)	494 (21.49)		11 834 (4.1)	11 515 (4.03)	319 (8.81)	
Hyperlipidemia				<0.001				<0.001
No	8895 (42.85)	8261 (43.82)	634 (33.61)		282 394 (97.71)	278 925 (97.74)	3469 (95.83)	
Yes	12 245 (57.15)	10 956 (56.18)	1289 (66.39)		6613 (2.29)	6462 (2.26)	151 (4.17)	
BMR				0.046				<0.001
Q1	5418 (24.98)	4977 (25.27)	441 (22.21)		72 242 (25.00)	71 737 (25.14)	505 (13.95)	
Q2	5718 (25.02)	5179 (25.13)	539 (23.88)		72 227 (24.99)	71 610 (25.09)	617 (17.04)	
Q3	5228 (25.00)	4755 (24.85)	473 (26.39)		72 335 (25.03)	71 245 (24.96)	1090 (30.11)	
Q4	4776 (25.00)	4306 (24.74)	470 (27.51)		72 203 (24.98)	70 795 (24.81)	1408 (38.90)	

BMI, body mass index; BMR, basal metabolic rate; KSD, kidney stone disease; SD, standard deviation; US NHANES, US National Health and Nutrition Examination Survey; WC, waist circumference; Q1 = lowest BMR quartile; Q4 = highest BMR quartile.

Descriptive characteristics of study participants stratified by KSD status (without KSD and with KSD) are presented. Continuous variables are shown as mean values (SD) and tested for significant differences using analysis of variance (ANOVA). Categorical variables are reported as frequencies (weighted percentages for US NHANES or percentages for UK Biobank) and tested for significant differences in distribution using the chi-square test.

When stratified by quartiles of BMR level, individuals with higher BMR tended to be younger and male. In the US NHANES, the mean age of participants in the highest BMR quartile was 40.57 years, compared to 52.76 years in the lowest quartile. The percentage of males was substantially higher in the higher BMR quartiles (Q1: 4.47% vs. Q4: 87.13%). Similar trends were observed in the UK Biobank cohort, where participants in the highest BMR quartile had a mean age of 54.28 years, significantly younger than those in the lowest quartile. Additionally, the percentage of males increased progressively across BMR quartiles, from 0.76% in Q1 to 97.73% in Q4.

### Initial discovery of the association between BMR and the risk of KSD in the US NHANES

Multivariable logistic regression models using the US NHANES cohort initially revealed a significant inverse association between BMR and the incidence of KSD (Table 2). A clear dose-response relationship was observed (*P* for trend < 0.001), with individuals in the highest BMR quartile (Q4) showing a substantially lower risk of developing KSD compared to those in the lowest quartile (Q1). Specifically, the OR for KSD in Q4 compared to Q1 was 0.49 (95% CI: 0.35, 0.70; *P* < 0.001) in the fully adjusted model (Model 3), indicating a 51% reduction in KSD risk among individuals with the highest BMR levels. These findings from NHANES provided initial evidence of a protective effect of higher BMR against KSD.

**Table 2:** Associations of BMR with incident KSD based on KSD status

	Model 1 OR (95% CI)	Model 2 OR (95% CI)	Model 3 OR (95% CI)
US NHANES			
BMR Q1	Reference	Reference	Reference
BMR Q2	0.66(0.54,0.81) ***	0.75(0.60,0.93) **	0.80(0.64,0.99) *
BMR Q3	0.50(0.39,0.65) ***	0.64(0.48,0.84) **	0.70(0.53,0.92) *
BMR Q4	0.30(0.23,0.39) ***	0.43(0.31,0.60) ***	0.49(0.35,0.70) ***
*P* for trend	**<0.001**	**<0.001**	**<0.001**
UK Biobank			
BMR Q1	Reference	Reference	Reference
BMR Q2	0.80(0.70,0.91) ***	0.82(0.72,0.93) **	0.82(0.72,0.94) **
BMR Q3	0.74(0.63,0.88) ***	0.78(0.66,0.93) **	0.80(0.67,0.94) **
BMR Q4	0.65(0.53,0.79) ***	0.70(0.57,0.86) ***	0.72(0.59,0.89) **
*P* for trend	**<0.001**	**<0.001**	**0.003**

BMR, basal metabolic rate; CI, confidence intervals; KSD, kidney stone disease; OR, odds ratio; US NHANES, US National Health and Nutrition Examination Survey.

The model outcome was KSD status (binary: without KSD and with KSD). Quartiles (Q1–Q4) were categorized based on BMR quartiles in the US NHANES and UK Biobank cohorts. The analysis in the US NHANES cohort included the US population and study design weights to account for the complex survey design. Only participants free from KSD at baseline were included in the analysis for incident KSD in the UK Biobank cohort. Model 1: Adjusted for body mass index, waist circumference, and sex. Model 2: Adjusted for body mass index, waist circumference, sex, age, race, education, and income level. Model 3: Adjusted for body mass index, waist circumference, sex, age, race, education level, income level, smoking status, alcohol consumption, sleep duration, physical activity, hypertension, diabetes, and hyperlipidemia. Significance levels: ***P < 0.001, **P < 0.01, *P < 0.05.

### Validation of causal evidence in UK Biobank

To further validate the association observed in the US NHANES and assess its causal nature, we analyzed the UK Biobank cohort. Consistent with NHANES findings, data from the UK Biobank also demonstrated an inverse causal association between BMR and KSD risk (Table 2). In the fully adjusted model (Model 3), individuals in the highest BMR quartile (Q4) had an OR of 0.72 (95% CI: 0.59, 0.89; *P* for trend = 0.003) compared to those in the lowest quartile (Q1), representing a 28% reduction in KSD risk. The consistency of these results across two large, independent cohorts reinforces the potential causal relationship between higher BMR and reduced KSD risk, underscoring the importance of metabolic activity in KSD prevention.

### Dose-response relationship between BMR and KSD

**Figure 1.**
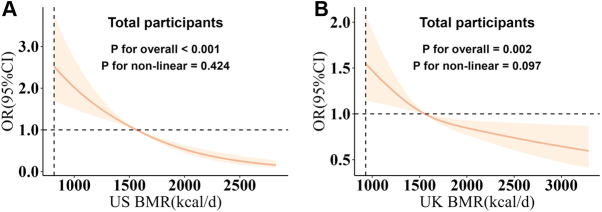
Dose-response relationship between basal metabolic rate (BMR) and the risk of kidney stone disease (KSD). Restricted cubic spline (RCS) models depict the non-linear relationship between BMR and KSD risk in the total participants from the US National Health and Nutrition Examination Survey (US NHANES) (Panel A) and UK Biobank (Panel B) cohorts. Odds ratios (OR) were adjusted for sex, age, race, education level, income level, smoking status, alcohol consumption, sleep duration, physical activity, body mass index, waist circumference, and prevalent comorbidities (including a history of hypertension, diabetes, and hyperlipidemia). The solid lines represent the adjusted OR for KSD risk across the BMR distribution, and the shaded areas represent the 95% confidence intervals (CI). The reference value (OR = 1) is set at the median BMR. The *P* value for non-linearity indicates the significance of the non-linear association between BMR and KSD risk. (A) In the US NHANES, models incorporated US population and study design weights to account for the complex survey design. (B) In the UK Biobank, only participants free from KSD at baseline were included in the analysis for incident KSD.

Building on the findings from the logistic regression models, we conducted RCS analyses to further characterize the dose-response relationship between BMR and KSD incidence (Fig. 1). In the US NHANES cohort, a significant linear inverse association was observed (*P* for non-linearity = 0.424; Fig. 1A), indicating a stable reduction in KSD risk across the BMR spectrum. In the UK Biobank cohort, a similar pattern was observed, although the relationship showed a slight tendency to flatten at higher BMR levels (*P* for non-linearity = 0.097; Fig. 1B). These results provide additional evidence of a potential dose-dependent protective effect of BMR against KSD.

### Subgroup analysis

**Figure 2.**
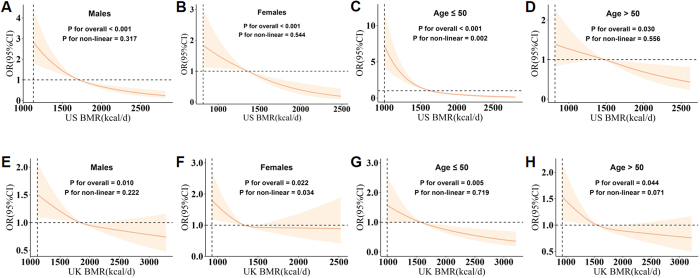
Subgroup analysis of the association between basal metabolic rate (BMR) and the risk of kidney stone disease (KSD) by sex and age. Sex: Panels A and B for NHANES; Panels E and F for UK Biobank. Age (≤50 and >50 years): Panels C and D for NHANES; Panels G and H for UK Biobank. Odds ratios (OR) were adjusted for sex, age, race, education level, income level, smoking status, alcohol consumption, sleep duration, physical activity, body mass index, waist circumference, and prevalent comorbidities (including a history of hypertension, diabetes, and hyperlipidemia). The solid lines indicate the adjusted OR for KSD risk across BMR levels, with the shaded areas representing 95% confidence intervals (CI). The *P* values for non-linearity assess the presence of non-linear associations in each subgroup.

To investigate whether the observed association varied across specific subpopulations, subgroup analyses were performed, stratified by sex (male, female) and age (≤50 years, >50 years) (Fig. 2). The overall trend showed a consistent inverse association between BMR and KSD risk across all subgroups, with higher BMR linked to a lower risk of KSD in both the US NHANES and UK Biobank cohorts (Fig. 2A–2H). However, variations in the strength and pattern of this association were observed across sexes and age groups.

**Figure 3.**
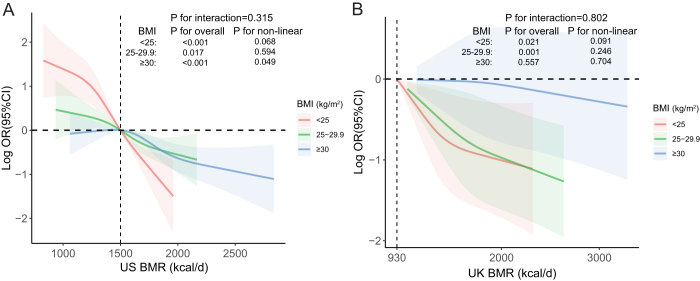
BMI subgroups and interaction analysis of the association between basal metabolic rate (BMR) and the risk of kidney stone disease (KSD). Panel A represents data from the US NHANES, while B represents data from the UK Biobank. Odds ratios (OR) were adjusted for sex, age, race, education level, income level, smoking status, alcohol consumption, sleep duration, physical activity, body mass index, waist circumference, and prevalent comorbidities (including a history of hypertension, diabetes, and hyperlipidemia). The solid lines indicate the adjusted OR for KSD risk across BMR levels, with the shaded areas representing 95% confidence intervals (CI). The *P* values for non-linearity assess the presence of non-linear associations in each subgroup. *P* for interaction evaluates the interaction between BMI and BMR in both cohorts. Data are presented for BMI categories: <25, 25–29.9, and ≥30.

Among males, a largely linear dose-response pattern was observed in both cohorts (Fig. 2A, US NHANES; *P* for overall < 0.001; *P* for non-linearity = 0.317) (Fig. 2E, UK Biobank; *P* for overall = 0.010; *P* for non-linearity = 0.222). In females from the UK Biobank cohort, the effect of BMR on KSD risk was more pronounced at lower BMR levels but plateaued at higher values (Fig. 2F, UK Biobank; *P* for overall = 0.022; *P* for non-linearity = 0.034). In younger individuals (Fig. 2C and 2G), the inverse association between BMR and KSD risk was more dynamic, with a sharp reduction in KSD risk at lower BMR levels, which plateaued as BMR increased (Fig. 2C, US NHANES; *P* for overall < 0.001; *P* for non-linearity = 0.002). In contrast, older individuals (Fig. 2D and 2H) exhibited a more linear and moderate reduction in KSD risk with increasing BMR. As shown in Fig. 3, interaction analyses based on BMI categories (<25, 25–29.9, and ≥30) were performed in both the US NHANES and UK Biobank cohorts. In the US NHANES cohort, BMR was inversely associated with developing KSD in all three groups (*P* for overall < 0.05), of which this inverse association was more stable after BMR≥1500 kcal/d in participants with BMI≥30 (P for non-linearity = 0.049). In the UK Biobank cohort, similar trends were observed in the BMI < 25 and 25 ≤ BMI < 30 groups (*P* for overall < 0.05), but not in the BMI ≥ 30 group (*P* for overall = 0.557). However, no significant interaction was observed across the BMI groups in either cohort (*P* for interaction = 0.315 for US NHANES and *P* for interaction = 0.802 for UK Biobank). These subgroup analyses deepen our understanding of how the protective effect of BMR may vary across different populations, emphasizing the potential of BMR as a modifiable factor in KSD prevention, particularly among high-risk subpopulations.

### Sensitivity analysis

**Figure 4.**
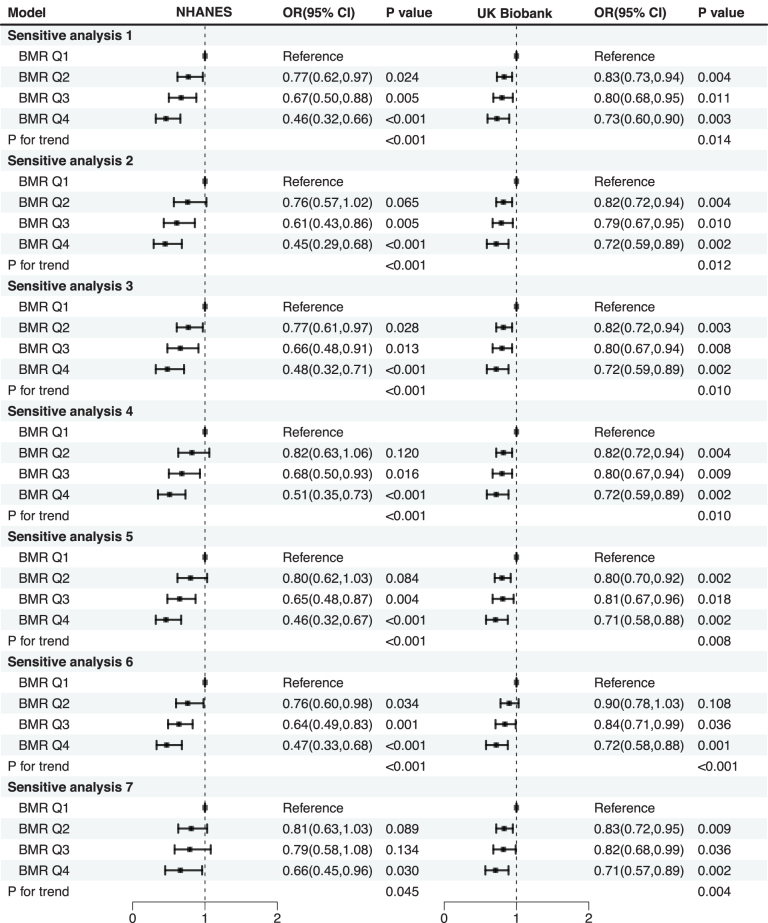
Sensitivity analysis based on fully adjusted model 3 for the association between BMR and KSD risk in the NHANES and UK Biobank cohorts. The model outcome was KSD status (binary: without KSD and with KSD). Quartiles (Q1–Q4) were categorized based on the BMR quartiles in the US NHANES and UK Biobank cohorts. Sensitive analysis 1: Excluded patients with baseline renal failure. Sensitive analysis 2: Excluded patients with baseline hypercalcemia and increased levels of vitamin D. Sensitive analysis 3: Excluded patients with baseline hyperuricemia or gout. Sensitive analysis 4: Excluded patients with baseline thyroid disease. Sensitive analysis 5: Excluded patients with baseline tumors. Sensitive analysis 6: Used the Harris-Benedict formula to recalculate BMR and excluded patients with extreme ages, heights, and weights in both US NHANES and UK Biobank. Sensitive analysis 7: Excluded patients diagnosed with KSD within 3 years of follow-up and further adjusted for renal failure, hypercalcemia, increased levels of vitamin D, hyperuricemia/gout, thyroid disease, and tumor history (UK Biobank only); restricted participants aged 37–73 years (US NHANES only). Abbreviations: BMR, basal metabolic rate; CI, confidence intervals; KSD, kidney stone disease; OR, odds ratio; US NHANES, US National Health and Nutrition Examination Survey.

To further validate the robustness of the observed association in the US NHANES and evaluate its causal nature in the UK Biobank, multiple sensitivity analyses were conducted with various exclusion criteria (Fig. 4). In each sensitivity analysis, the fully adjusted Model 3 was applied separately with different exclusion criteria, including the removal of participants with baseline renal failure (sensitivity analysis 1), hypercalcemia and increased levels of vitamin D (sensitivity analysis 2), gout or hyperuricemia (sensitivity analysis 3), thyroid disease (sensitivity analysis 4), malignancy (sensitivity analysis 5), as well as recalculating BMR using another internationally recognized equation and excluding participants with extreme anthropometric values (sensitivity analysis 6). Across all these conditions, the inverse causal association between higher BMR and lower KSD risk remained consistent. For instance, in sensitivity analysis 7, after excluding patients diagnosed with KSD within 3 years of follow-up and additionally adjusting for baseline renal failure, hypercalcemia, increased levels of vitamin D, gout or hyperuricemia, thyroid disease, and tumor history in the UK Biobank, the fully adjusted OR for KSD in the highest BMR quartile (Q4) compared to the lowest quartile (Q1) was 0.71 (95% CI: 0.57, 0.89; *P* = 0.002), indicating a 29% reduction in KSD risk. The trend test remained significant (*P* for trend = 0.004), further reinforcing the robustness of the BMR-KSD association. Similarly, when restricting the age range to 37–73 years in the US NHANES cohort, the results remained consistent, with an OR of 0.66 (95% CI: 0.45, 0.96; *P* = 0.030; *P* for trend = 0.045). Overall, these consistent results across sensitivity analyses further underscore the potential protective role of a higher metabolic rate against kidney stone formation, suggesting that a higher BMR may independently contribute to a lower risk of KSD.

## Discussion

This study provides compelling evidence of a significant negative causal relationship between basal metabolic rate and the risk of kidney stone disease, from the US to the UK cohorts. Our results suggest that low BMR can increase the risk of developing KSD, even after adjusting for a broad range of potential confounding factors, including demographic variables, lifestyle factors, and metabolic comorbidities. These findings remained consistent across various sensitivity analyses and demographic subgroups, including age and sex.

The inverse relationship between BMR and KSD risk observed in our study suggests potential biological mechanisms that warrant further exploration. BMR reflects overall energy expenditure, which may influence metabolic processes involved in kidney stone formation. Specifically, higher BMR is often correlated with more frequent physical activity and healthier lifestyle behaviors^[4,38,39]^, both of which could reduce risk factors for kidney stone formation, such as obesity, insulin resistance, and oxidative stress^[40]^. Physical activity has been shown to improve urinary calcium oxalate crystal excretion and enhance renal health, thereby preventing the accumulation of stone-forming materials^[41]^. Additionally, kidneys require sufficient energy expenditure to maintain normal urine excretion, including the removal of solutes such as calcium, oxalate, and uric acid^[42]^. Increased energy expenditure may affect urinary excretion patterns, potentially reducing the urinary concentrations of stone-forming substances like calcium and oxalate^[43]^. In contrast, a decrease in metabolic rate may impair the kidneys’ ability to efficiently excrete these minerals, leading to their accumulation in the urine and promoting stone formation^[11]^. Lower BMR can result in reduced energy expenditure, which is likely related to metabolic imbalances^[7,44]^, recognized as a risk factor for kidney stone formation, particularly because energy imbalance disrupts metabolic processes^[42,43]^. BMR plays a critical role in maintaining energy balance, and when it declines, the body’s ability to regulate energy consumption also decreases, potentially exacerbating metabolic disorders^[38,44]^. These disorders, including obesity, diabetes, and hypertension, increase the risk of kidney stones by promoting the buildup of stone-forming substances such as calcium oxalate and calcium phosphate in the urine^[11,20,32,43]^. Therefore, maintaining an adequate BMR is essential for preventing metabolic imbalance and the subsequent development of kidney stones. Future research should focus on elucidating these mechanisms to better understand the protective role of higher BMR in KSD prevention.

Overall, BMR is inversely associated with KSD across all subgroups, but the strength and pattern of this association vary by sex and age. These findings suggest potential sex- and age-related differences in the metabolic processes that influence kidney stone formation. Both males and females benefit from higher BMR in terms of reduced KSD risk, but males exhibited a more linear and stable protective effect, with a consistent decrease in risk as BMR increased. In contrast, females, particularly in the UK Biobank cohort, displayed a more complex, non-linear relationship. In this group, the protective effect of BMR was stronger at lower BMR values but less pronounced as BMR increased. This non-linear association may be related to sex-specific metabolic and hormonal differences, which can influence both BMR levels and kidney stone formation. Women, especially premenopausal women, are known to have lower rates of KSD compared to men, which may be partly attributed to hormonal regulation and calcium metabolism^[39,45]^. Our findings support the notion that metabolic factors such as BMR may contribute to these observed sex differences. Age-related changes in renal function and metabolic rate suggest that BMR tends to decline with age, primarily due to the loss of muscle mass^[3,38]^, which may further impair the kidneys’ ability to efficiently excrete stone-forming substances^[42,43,45]^. This age-related difference suggests that younger populations might benefit more from interventions aimed at increasing BMR, whereas in older adults, the protective effect of BMR may be limited by age-related metabolic decline. This means that the elderly need to take more measures to maintain or improve BMR^[46]^. Besides, our results suggest that interventions aimed at maintaining or increasing metabolic activity in younger populations may provide long-lasting protection against the development of kidney stones later in life^[10]^. These findings highlight the importance of considering both sex and age when evaluating the protective role of BMR in KSD prevention, suggesting that different strategies may be required to optimize BMR influence on KSD in various demographic groups.

Our findings align with previous research that has highlighted metabolic health and energy balance as important factors in the development of kidney stones^[11]^. However, to our knowledge, this is the first large-scale study to specifically investigate the relationship between BMR and KSD risk across diverse populations. While prior studies have primarily focused on individual metabolic factors, such as obesity, insulin resistance, and dietary intake^[47]^, our results suggest that BMR – a comprehensive indicator of metabolic activity – may serve as a novel predictor of KSD risk. A previous retrospective study of more than 300 obese patients diagnosed with KSD by Kang *et al* found that BMR was positively correlated with the recurrence of urolithiasis in obese patients^[21]^. However, the study merely reported that obese patients with higher BMR were more prone to recurrent urolithiasis, without providing sufficient evidence to directly link high BMR with an increased risk of kidney stones. The observed correlation may have been confounded by obesity. More importantly, as a single-center retrospective study, it was limited by small sample size, cross-sectional design, and lack of generalizability. To minimize the interference of confounding factors, we conducted a two-sample Mendelian randomization (MR) analysis in our previous study to assess the causal relationship between BMR and the risk of kidney stones, yielding results that contradicted earlier observational studies^[22]^. Since genetic variation is randomly distributed, MR analysis can provide more accurate results than traditional observational studies by reducing potential bias^[48]^. Similarly, our preliminary genome-wide association study (GWAS) analyzed the relationship solely from a genetic perspective and lacked direct validation at the individual level. Therefore, the current study aims to further investigate the causal relationship between BMR and KSD from a clinical etiological perspective across global populations and to elucidate their dose-response relationship. In contrast, our study utilizes large-scale, population-based data from both the US NHANES and the UK Biobank cohorts, which include diverse populations with varying BMI categories. By leveraging these extensive datasets, we investigate the relationship between BMR and KSD risk across a broader population, beyond just obese individuals, with comprehensive epidemiological methods, including dose-response analyses, sensitivity testing, and robust adjustments for a wide range of confounding factors. Our prospective study design helps mitigate reverse causality concerns, further enhancing the credibility of the findings. Notably, our findings demonstrate that higher BMR is independently linked to a reduced risk of kidney stones, even after adjusting for BMI and waist circumference, suggesting that the protective effect of higher BMR may involve mechanisms beyond body weight alone. The cross-cohort consistency of our findings, despite demographic and regional differences, strengthens the potential generalizability of BMR as a protective factor against KSD, offering clinically relevant evidence for future prevention strategies.

The identification of BMR as a potential protective factor for KSD risk has important clinical implications. First, BMR could be considered a novel risk factor for kidney stone formation, independent of traditional factors such as BMI and waist circumference. Routine BMR assessments could help identify individuals at higher risk of KSD, facilitating early intervention. Second, interventions aimed at increasing BMR, such as promoting physical activity and optimizing metabolic health, could be incorporated into KSD prevention strategies, particularly for high-risk populations^[10,18,19,40,49]^. However, further studies are required to establish standardized BMR thresholds and to explore how lifestyle modifications that elevate BMR impact KSD risk in various subpopulations.

While our study has several strengths, including large sample sizes, cross-cohort validation, comprehensive adjustment for potential confounders, and rigorous sensitivity analyses, there are some limitations to consider. First, BMR was estimated rather than directly measured. Although predictive formulas like the Mifflin-St Jeor equation are widely used and convenient for large-scale studies, they may not accurately capture individual variations in metabolism compared to direct or indirect calorimetry^[50]^. Additionally, estimating BMR based on anthropometric data may introduce measurement error, although sensitivity analyses excluding extreme values and recalculating BMR produced consistent results. Second, while we adjusted for a wide range of confounders, residual confounding from unmeasured factors, such as dietary intake of specific nutrients related to stone formation, may still exist^[18]^. Moreover, some key risk factors for urolithiasis, such as hyperparathyroidism, mineral bone disorder, nephrocalcinosis, polycystic kidney disease (PKD), and gastrointestinal diseases, were not included in the analysis due to data constraints in the NHANES and UK Biobank databases^[51,52,53]^. Lastly, the cross-sectional nature of the US NHANES dataset limits our ability to infer causality. Although the UK Biobank provides prospective data, the follow-up period may not fully capture the long-term development of KSD^[54]^. Further research is needed to elucidate the specific biological mechanisms underlying this causal relationship and how it manifests across different populations. Longitudinal studies with more detailed dietary and metabolic data could provide insights into how changes in BMR over time influence kidney stone risk. Additionally, intervention studies aimed at increasing metabolic activity through exercise or dietary modifications could help determine whether enhancing BMR can effectively reduce the incidence of kidney stones in high-risk populations.

## Conclusion

In conclusion, our study demonstrates that higher BMR is causally associated with a lower risk of KSD, from the initial discovery in the US NHANES cohort to the validation in the UK Biobank cohorts. This suggests that BMR could serve as an individualized factor in the clinical assessment and management of KSD risk, potentially aiding in the development of strategies to prevent recurrence. These findings offer a novel perspective on KSD prevention, emphasizing the importance of metabolic health and energy expenditure.

## Data Availability

The NHANES datasets are publicly available at https://wwwn.cdc.gov/nchs/nhanes/default.aspx. The UK Biobank datasets are available at https://www.ukbiobank.ac.uk, subject to a registration and application process (Application number: 143798).

## Research registration unique identifying number (UIN)

This study was conducted in accordance with the principles of the Declaration of Helsinki and was registered in the Research Registry (https://www.researchregistry.com/) under the unique identifying number: researchregistry11226.
